# Preclinical PET Imaging of Granzyme B Shows Promotion of Immunological Response Following Combination Paclitaxel and Immune Checkpoint Inhibition in Triple Negative Breast Cancer

**DOI:** 10.3390/pharmaceutics14020440

**Published:** 2022-02-18

**Authors:** Tiara S. Napier, Chanelle L. Hunter, Patrick N. Song, Benjamin M. Larimer, Anna G. Sorace

**Affiliations:** 1 Graduate Biomedical Sciences Cancer Biology, University of Alabama at Birmingham, Birmingham, AL 35294, USA; tnapier@uab.edu (T.S.N.); hunterc1@uab.edu (C.L.H.); psong@uabmc.edu (P.N.S.); 2 Department of Radiology, University of Alabama at Birmingham, Birmingham, AL 35294, USA; blarimer@uab.edu; 3 O’Neal Comprehensive Cancer Center, University of Alabama at Birmingham, Birmingham, AL 35294, USA; 4 Department of Biomedical Engineering, University of Alabama at Birmingham, Birmingham, AL 35294, USA

**Keywords:** PET, GZP-PET, tumor microenvironment, immunotherapy, anti-PD-1, anti-CTLA4, 4T1, E0771

## Abstract

Advancements in monitoring and predicting of patient-specific response of triple negative breast cancer (TNBC) to immunotherapy (IMT) with and without chemotherapy are needed. Using granzyme B-specific positron emission tomography (GZP-PET) imaging, we aimed to monitor changes in effector cell activation in response to IMT with chemotherapy in TNBC. TNBC mouse models received the paclitaxel (PTX) ± immune checkpoint inhibitors anti-programmed death 1 (anti-PD1) and anti-cytotoxic T-lymphocyte 4 (anti-CTLA4). GZP-PET imaging was performed on treatment days 0, 3, and 6. Mean standard uptake value (SUV_mean_), effector cell fractions, and SUV histograms were compared. Mice were sacrificed at early imaging timepoints for cytokine and histological analyses. GZP-PET imaging data revealed differences prior to tumor volume changes. By day six, responders had SUV_mean_ ≥ 2.2-fold higher (*p* < 0.0037) and effector cell fractions ≥ 1.9-fold higher (*p* = 0.03) compared to non-responders. IMT/PTX resulted in a significantly different SUV distribution compared to control, indicating broader distribution of activated intratumoral T-cells. IMT/PTX resulted in significantly more necrotic tumor tissue and increased levels of IL-2, 4, and 12 compared to control. Results implicate immunogenic cell death through upregulation of key Th1/Th2 cytokines by IMT/PTX. Noninvasive PET imaging can provide data on the TNBC tumor microenvironment, specifically intratumoral effector cell activation, predicting response to IMT plus chemotherapy.

## 1. Introduction

Triple negative breast cancer (TNBC) represents up to 20% of all breast cancers and is associated with poorer prognosis compared to other breast cancer subtypes [1,2,3]. TNBC is characterized by high risk of recurrence, short-term progression-free survival, and the worst breast cancer-specific survival [4,5]. Additionally, at the time of patient diagnosis, histological reports often detect TNBC tumors when they are high grade (i.e., infiltrating ductal carcinomas) [3]. Intra- and intertumoral heterogeneity combined with a lack of estrogen receptors, progesterone receptors, and low human epidermal growth factor receptor 2 (HER2) increases the challenge of identifying effacicious targets for personalized therapy. Only a portion of tumors respond to antimitotic agents such as paclitaxel (PTX) [1]; therefore, due to success in clinical trials, immunotherapy (IMT) is now included as standard-of-care in early-stage, high-risk TNBC patients. While there has been some success, immune checkpoint blockades with programmed death 1 (PD-1) and cytotoxic T-lymphocyte-associated protein 4 (CTLA-4) inhibitors have not proven effective for all TNBC patients [6,7]. Due to the limited responsivity observed with single-agent therapies, ongoing clinical studies are evaluating the use of novel TNBC treatment regimens that combine FDA-approved chemotherapeutic agents with IMT [8]. Current preclinical efforts focus on developing IMT combinations that convert nonresponding solid tumors to responders, deepening tumor response, and overcoming acquired resistance to IMT could guide clinical trials. Insights into the molecular profile of responsive tumors versus non-responsive tumors would allow precise, cost-effective treatment of TNBC.

It is understood that some breast cancers are immunogenic and that the tumor microenvironment (TME) plays an important role in shaping response to various anti-cancer therapies, including IMT [9,10]. Compared to other breast cancer subtypes, TNBC tumors have higher immunogenicity, higher enrichment by tumor-infiltrating lymphocytes (TILs), and higher levels of programmed cell death ligand 1 (PD-L1) expression [1]. Additionally, the TME has been shown to be involved in PD-1 regulation, cytokine secretion, and the promotion of various immune cell populations [11]. Due to immune checkpoint blockades showing signs of success in TNBC [12,13], several studies have examined the use of immune checkpoint antibody inhibitors to regulate T cell populations and activities in the TME [14,15,16,17]. Preclinical studies reveal that dual checkpoint blockade with anti-PD1 and anti-CTLA4 decreases replacement of T effector cells with T regulatory cells, enhances dendritic cell activation, and increases activation of tumor-infiltrating cytotoxic CD8^+^ and CD4^+^ T cells [14,16]. Recently, granzyme B, the serine protease downstream effector of cytotoxic T cells, has been shown to serve as a useful predictive biomarker for efficacious responses to IMT [18,19,20]. Despite numerous efforts in understanding the role of immune activation during immunotherapy response in TNBC, many preclinical studies focus on immunotherapy alone. However, numerous clinical trials are utilizing it in conjunciton with other treatments, which can alter the underlying biology of the tumor.

The majority of breast cancer clinical trials examining checkpoint inhibition have been conducted in TNBC, in large part because of its immunogenic phenotype [14]. Wide variations in response kinetics have been observed in immunotherapy treatment compared to traditional cytotoxic therapy (chemotherapies and radiation) [21]. This variation makes assessment monitoring or predictive imaging on a personalized basis difficult [22]. Traditional anatomical imaging techniques and Response Evaluation Criteria in Solid Tumours (RECIST; v.1.1) do not accurately reflect the biological and immunological changes within the tumor during IMT [23,24,25]. Therefore, there is a great need to quantitatively assess tissue-scale changes that occur during IMT. Positron emission tomography (PET) imaging provides quantifiable information at the molecular level that describes the underlying biology and enables measurement and tracking of cellular signaling early in the course of anti-cancer treatments [25]. Currently, TNBC has no clinically validated molecular imaging biomarkers; however, PET with [^18^F]-fluorodeoxyglucose (FDG), a non-specific biomarker of glycolytic metabolism, is often used to detect metabolically active malignant lesions [26]. Limitations of FDG-PET include specificity, accurate identification of pseudoprogression and hyperprogression, and the ability to distinguish immune-related inflammation from progression due to treatment with IMT [27]. Rather than relying on glucose metabolism as a tumor biomarker, recent studies provide evidence in support of specific immunoPET techniques that target overexpressed proteins such as CD8 and PD-L1, which are expressed in the TME and enhanced during IMT [28,29,30]. ImmunoPET techniques target proteins that are key players in the mechanisms of IMT and are more specific to immune and cancer cells than glycolytic metabolism [31,32,33]. Analyzing the TME for reliable predictive biomarkers of tumor status represents a viable approach since the TME can greatly influence response to therapy [10,34,35,36].

In this contribution, we examine the relationship between effector cell activation and response to the combination of IMT and chemotherapy in two syngeneic mouse models of TNBC. To do this, we use advanced PET imaging with a granzyme B radiotracer to predict immune response in TNBC tumors early in the course of treatment and quantified whether it can be utilized as a predictive biomarker of response. The three-dimensional information provided by granzyme B based-PET (GZP-PET) imaging, a novel technology enabling a global view of effector cell activation, allows noninvasive visualization of the tumor microenvironment that can be used to assess immune response over time [18,19,37,38,39]. Quantitative assessment of the percent of tumor that has positive effector cell activation and the range of tumor heterogeneity of activation with GZP-PET represents a viable and not yet undertaken approach in evaluating immune checkpoint blockade efficacy in combination with chemotherapy for TNBC. Furthermore, as combination therapies are common in TNBC, understanding the direct effects of chemotherapy on granzyme B and effector cell activation could help guide treatment regimens that utilize IMT. This advanced imaging technique offers the potential to minimize bias observed in tumor biopsies, which only assess information from a single point in time and provide only a small sample section of the tumor. GZP-PET is an advanced, yet clinically translatable imaging approach that has the potential to identify predictive biomarkers of treatment response to combination chemotherapy and IMT and may be utilized to help understand which chemotherapeutic treatments used in combination with IMT will be effective for individual tumors in TNBC [18,40].

## 2. Materials and Methods

### 2.1. Cell Culture

Triple-negative murine mammary carcinoma 4T1, cells were purchased from American Type Culture Collection (Manassas, VA, USA). The 4T1 cells were transduced with cytomegalovirus-luciferase and cultured in 10% fetal bovine serum in Roswell Park Memorial Institute 1640 complete growth media (Gibco, Waltham, MA, USA). Triple-negative murine mammary carcinoma E0771 cells were purchased from CH3 Biosystems (Buffalo, NY, USA) and cultured in 10% fetal bovine serum in Dulbecco’s Modified Eagle Medium (Gibco) with 2 mM L-glutamine and 1 mM sodium pyruvate. All cells were cultured in a humidified incubator at 37 °C with 5% CO_2_, grown to 80% confluence, and maintained at passage numbers less than 20. All experiments were performed with cells acquired and frozen within one month to maintain the phenotype of each cell line.

### 2.2. Syngeneic Mouse Tumor Models

All procedures involving animals were reviewed and approved by the Institutional Animal Care and Use Committee (IACUC) at the University of Alabama at Birmingham (IACUC protocol number: 08778). 2 × 10^5^ 4T1 cells in 100 µL phosphate buffered saline (PBS) were orthotopically injected into the 3rd mammary fat pad of five to six week old female Balb/c mice (Charles River Laboratories, Wilmington, MA, USA). 5 × 10^5^ E0771 cells in 100 µL phosphate buffered saline containing 40% Matrigel were orthotopically injected into the 3rd mammary fat pad of 10–12 week old female C57Bl6 mice (Jackson Laboratory, Bar Harbor, ME, USA). Primary tumor volumes were calculated with calipers using the formula: $V=\left(\frac{4\pi }{3}\right)\times \left[\frac{shortest\;diameter\;x\;longest\;diameter\;x\;average\;of\;shortest\;and\;longest\;diameters)}{2}\right].$ When 4T1 tumors (*n* = 60) reached 80–100 mm^3^ and E0771 tumors (*n* = 37) reached 75–300 mm^3^, mice were enrolled into the study. All mouse procedures and care were completed in accordance with the protocols approved by IACUC.

### 2.3. Treatments

**Figure 1:**
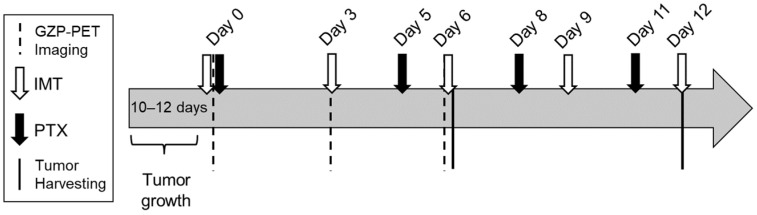
Experimental timeline. Abbreviations: Granzyme B specific positron emission tomography (GZP-PET), immunotherapy (IMT), paclitaxel (PTX).

Paclitaxel (10 mg/kg; Alfa Aesar, Ward Hill, MA, cat. J62734-MC), anti-PD1 (200 μg), and anti-CTLA4 (100 µg) (Bio X Cell, Lebanon, NH, USA, cat. BE0146 and BE0164, respectively) were administered intraperitoneally with a total injection volume of 100 μL per mouse. Treatments were given as shown in Figure 1. For both tumor models, response to treatment was determined with a threshold calculation using the mean tumor volume of the control group on day 30 minus one standard deviation. There were four cohorts of animals per model, and animals were enrolled into long-term treatment response, imaging, or biological validation studies.

### 2.4. NOTA-GZP Synthesis and ^68^Ga Radiolabeling

NOTA–Ala–Gly–Gly–Ile–Glu–Phe–Asp–CHO (NOTA-GZP) and biotin–β-Ala–Gly–Gly–Ile–Glu–Pro–Asp–CHO (hGZP) were synthesized and analyzed for chemical purity as previously described [18]. Gallium 68 (^68^Ga) was obtained from a ^68^Ge/^68^Ga generator (iThemba Labs) eluted with 0.1 M HCl. Eluent was equilibrated to pH 4.5 with 1M sodium acetate followed by the addition of 50 μg NOTA-GZP. The dose of the radiolabeling is chosen to ensure that no interference with the functionality of granzyme B is introduced [19]. The labeling reaction proceeded at 37 °C while shaking at 300 rpm for 10 min. The product was loaded on a reverse-phase C18 Sep-Pak mini cartridge and eluted with 200 μL of 70% ethanol in dPBS. NOTA-GZP affinity and specificity determination was performed as previously described [18]. Specific activity was assessed by gamma counter and utilized if >95% purity.

### 2.5. GZP-PET/CT Imaging and Analysis

Tumor-bearing mice (*n* = 28 for E0771, *n* = 16 for 4T1) received 8.35 ± 1.85 MBq per mouse of NOTA-GZP bound to ^68^Ga in a total injection volume of 100–200 µL sterile saline. Dose was allowed to circulate systemically for 60 min and then a 20 min static GZP-PET image was performed and followed by computed tomography (CT) scanning on a GNEXT microPET/CT (SOFIE, Culver City, CA, USA) for mice before treatment on days 0, 3, and 6 as shown in Figure 1. PET and CT images were registered, and the whole tumor region of interest (ROI) was manually drawn based on the anatomical CT images. ROIs were reviewed by someone with >10 years’ experience in preclinical cancer imaging and cancer models. Mean and frequency histogram of standardized uptake value (SUV) were quantified with VivoQuant pre-clinical image processing software (v.4.0; Boston, MA, USA). SUV was calculated as follows: $SUV=\frac{\begin{array}{c} Measured\;activity\;in\;an\;ROI\\ \left(Activity\;concentration\;in\;tissue\right) \end{array}}{\frac{Injected\;dose}{Body\;weight}}=\frac{MBq/\mathrm{mL}}{MBq/g}=g/\mathrm{mL}$. Background ROIs were drawn in the left atrium of each subject. Effector cell fraction was determined by quantifying the fraction of voxels in the tumor above background signal, which was designated as blood measured from the left atrium of the heart (0.01 for 4T1 tumors and 0.05 for E0771 tumors).

### 2.6. Determination of Th1 and Th2 Expressed Cytokine Levels in Serum

Serum levels of IFN-γ, IL-2, IL-4, and IL-12 were determined by using a ProcartaPlex multiplex immunoassay panel (ThermoFisher, Waltham, MA, USA, cat. EPX110-20820-901) and Luminex 200 (Bio-Rad, Hercules, CA, USA). E0771 tumor samples were collected and snap frozen in liquid nitrogen. Samples were homogenized in lysis buffer (ThermoFisher, cat. EPX-99999-000) and centrifuged at 13,000 rpm for 10 min at 4 °C. Supernatant was collected and total protein concentration was normalized between all samples. Each serum sample was tested in two magnetic bead wells in mouse ProcartaPlex panels. The net mean fluorescence intensity (MFI) was averaged for three samples per treatment group for a biological replicate of *n* = 3/group and technical replicate of *n* = 6/group.

### 2.7. Histological Analysis

Tumor samples were collected and fixed with 10% buffered formalin overnight at room temperature. Paraffin embedding and cutting of 5 µm-thick tumor slices were performed in the UAB Pathology Core Research Lab. Hematoxylin and eosin (H&E) staining was conducted as previously reported [41]. Immunohistochemical (IHC) analysis was performed to assess granzyme B expression. Primary antibodies for anti-granzyme B (dilution 1:100, R&D Systems, Minneapolis, MN, cat. AF1865) was incubated overnight at 4 °C. Negative controls for the immunostaining procedure were conducted by omission of the primary antibody. Analysis of positive IHC staining of the whole section was completed through automated custom MATLAB algorithms. Auto-segmentation, k-means clustering, and color thresholding was conducted as previously described and adapted for granzyme B specific staining based on positive control tissue [42]. Images were binarized for the summation of positively stained tissue pixels, and the percentage of positive staining was defined as: $\%\;positive\;staining=\frac{\;total\;no.\;positively\;stained\;pixels}{total\;no.\;tumor\;pixels\;with\;noise\;and\;background\;removed}\times 100$. For granzyme B stained tumor slice images, images were normalized for number of pixels and histograms of pixel count versus intensity were created to observe the distribution of pixel intensity, which allows for analysis of the heterogeneity of the stain across the tissue slice.

### 2.8. Statistics

Unpaired *t*-tests were used to compare non-responder and responder groups. Tumor volumes and SUV_mean_ from day zero to day six were compared with mixed ANOVA and Tukey’s post hoc test. Histogram plots were compared between treatment groups with Kolmogorov–Smirnov test. Spearman’s rank-order correlation was used for statistical analysis of correlation plots. A *p*-value of <0.05 was considered statistically significant. The following notations were used throughout: non-significant, *p* > 0.05; * *p* < 0.05; ** *p* < 0.01; *** *p* < 0.001; **** *p* <0.0001.

## 3. Results

### 3.1. PTX Alone and in Combination with IMT Increases Effector Cell Activation and Intratumoral Heterogeneity

**Figure 2:**
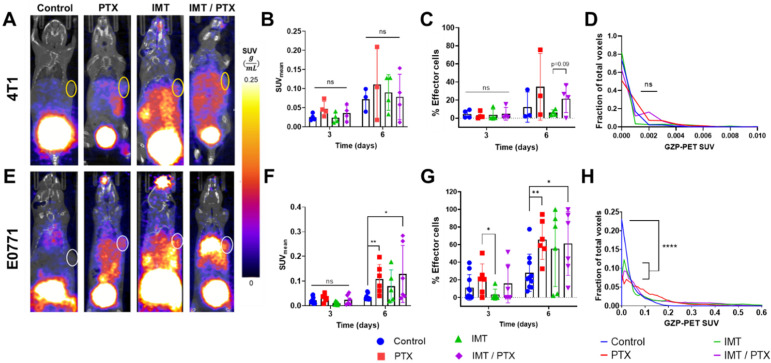
Granzyme B-specific positron emission tomography (GZP-PET) imaging provides quantitative data on triple negative breast cancer (TNBC) tumor response. Representative GZP-PET images on day six, mean standard uptake value (SUV_mean_), effector cell fraction, and histogram analysis of (**A**–**D**) 4T1 and (**E**–**H**) E0771 tumors. Combination immunotherapy (IMT)/paclitaxel (PTX)-treated TNBC tumors have higher radiotracer uptake, indicating higher effector cell activation, compared to control by day six. Mean ± SD (“ns” indicates not signficiant, *p* > 0.05, * indicates *p* < 0.05, ** indicates *p* < 0.01, **** indicates *p* < 0.0001; multiple *t*-tests).

GZP-PET images showed higher effector cell activation in IMT-treated TNBC tumors compared to control and PTX treated tumors by day six (Figure 2A,E). While no significant differences in SUV_mean_ of 4T1 tumors on days 3 and 6 were found (Figure 2B), the SUV_mean_ of E0771 tumors on day 6 was significantly higher in the PTX alone group (0.1077; *p* < 0.005) and in the combination IMT/PTX group (0.1288; *p* = 0.03) compared to control (0.0353; Figure 2F). No significant differences in the SUV_mean_ of livers in 4T1-tumor-bearing or E0771-tumor-bearing mice were found when treatment groups were compared to each other and control (*p* ≥ 0.10). Further quantitative analysis of GZP-PET images was performed to determine the percentage of effector cells in each tumor ROI. This analysis revealed that, by day 6, 4T1 tumors treated with combination IMT/PTX had a higher percentage of effector cells compared to treatment with IMT alone (23.1% vs. 3.9%; *p* = 0.09; Figure 2C).

In E0771 tumors, by day three, treatment with PTX alone resulted in significantly higher differences in the percent of effector cells compared to IMT alone (20.63% vs. 3.253%; *p* < 0.05; Figure 2G). Additionally, by day six, the percent of effector cells in E0771 tumors was significantly higher in the PTX alone group (65.59%; *p* < 0.005) and in the combination IMT/PTX group (61.19%; *p* = 0.03) compared to control (27.94%; Figure 2G). In addition to analysis of average effector cell activation per tumor, intratumoral distribution of effector cell activation was observed through histogram analysis of SUV per voxel of the tumor ROI. Histogram analysis compared the effect of individual and combination treatments on intratumoral heterogeneity. No significant changes in 4T1 tumor heterogeneity were found (Figure 2D); however, all treatments resulted in significantly broader distributions of SUV in E0771 tumors compared to control (*p* < 0.0001; Figure 2G). This result indicates that treatment with chemotherapy and IMT increases the distribution of effector cell activation in TNBC tumors.

### 3.2. GZP-PET Has Predictive Value for Response to IMT plus PTX

**Figure 3:**
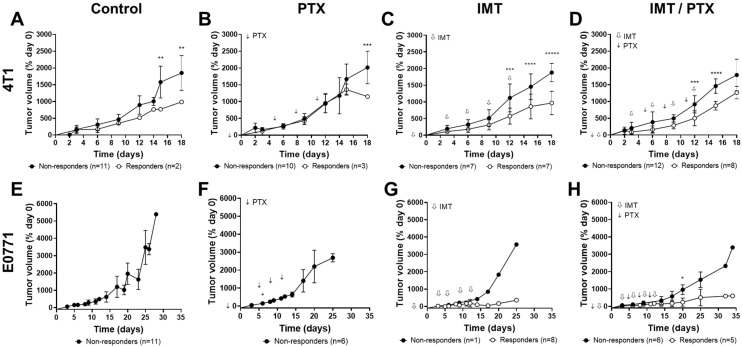
Mice were sorted into non-responder and responder groups based on tumor volume. Mice bearing (**A**–**D**) 4T1 and (**E**–**H**) E0771 tumors were separated into non-responder and responder groups for analysis of granzyme B-specific positron emission tomography predictive value. E0771 tumors showed better response to immunotherapy (IMT) ± paclitaxel (PTX) compared to 4T1 tumors. Mean ± SD (* indicates *p* < 0.05, ** indicates *p* < 0.01, *** indicates *p* < 0.001, **** indicates *p* < 0.0001, ***** indicates *p* < 0.00001; multiple *t*-tests).

To understand the predictive value of GZP-PET for therapeutic response, tumors were categorized as non-responders and responders using volumetric calculations of tumor size obtained through caliper measurements (Figure 3). Thresholds for response to treatment were determined using the mean growth of the control, saline-treated, mice. The earliest significant differences in tumor volume between responders and non-responders were observed on day 12 in the IMT alone and combination IMT/PTX groups.

Significant volumetric differences in non-responsive and responsive 4T1 tumors were observed on days 15 (*p* < 0.01) and 18 in PTX-treated 4T1 tumors (*p* < 0.01; Figure 3B). As noted in Figure 3A, two of the control tumors behaved differently than the rest, showing a small growth kinetic. 4T1 responders to IMT had significantly lower tumor volumes on days 12 (*p* < 0.005), 15 (*p* < 0.0005), and 18 (*p* < 0.000001) compared to non-responders (Figure 3C). Lastly, 4T1 responders to combination IMT/PTX had significantly lower tumor volumes on days 12 (*p* < 0.001) and 15 (*p* < 0.00005) compared to non-responders (Figure 3D). E0771 tumors treated with paclitaxel showed no changes in tumor volume compared to control (Figure 3E,F). E0771 responders to IMT showed lower tumor volumes on days 12–25 (Figure 3G). Significantly lower tumor volumes were found in E0771 responders to IMT/PTX by day 20 (*p* = 0.02; Figure 3H).

**Figure 4:**
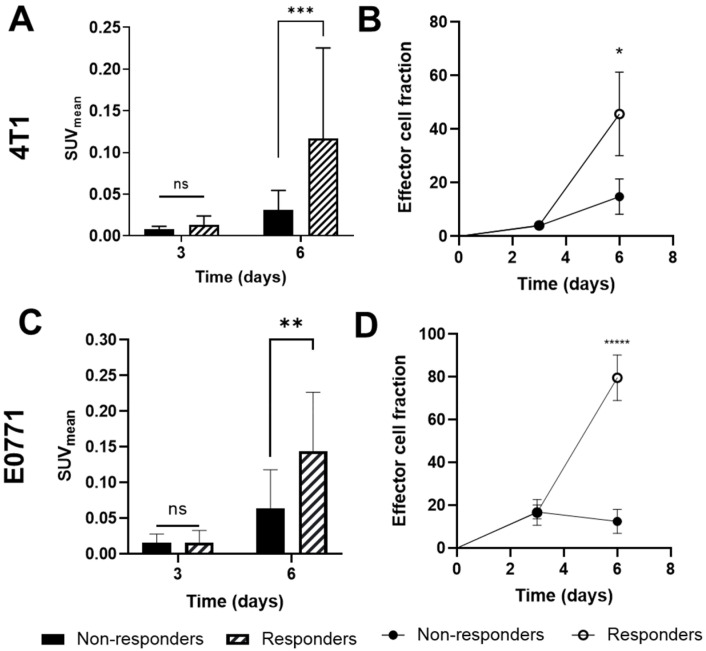
Granzyme B-specific positron emission tomography imaging data of effector cell activation accurately predicts response to immunotherapy (IMT) and chemotherapy in TNBC tumors. SUV_mean_ and effector cell fraction in (**A**,**B**) 4T1 (non-responders: *n* = 12; responders: *n* = 4) and (**C**,**D**) E0771 tumors (non-responders: *n* = 11; responders: *n* = 15). Mean ± SD (“ns” indicates not significant, *p* > 0.05, * indicates *p*  <  0.05, ** indicates *p* < 0.01, *** indicates *p* < 0.001, ***** indicates *p* < 0.00001; multiple *t*-tests).

SUV_mean_ and effector cell fraction derived from GZP-PET imaging data were analyzed to compare non-responding and responding 4T1 and E0771 tumors (Figure 4). By day six, in 4T1 tumors, SUV_mean_ was 3× higher (*p* = 0.03; Figure 4A) and effector cell fraction was 2× higher (*p* = 0.03; Figure 4B) in responding tumors compared to non-responding tumors. By day six, in E0771 tumors, SUV_mean_ was 2× higher (*p* < 0.005; Figure 4C) and effector cell fraction was 6× higher (*p* < 0.000001; Figure 4D) in responding tumors compared to non-responding tumors.

### 3.3. IMT Increases Necrosis and Granzyme B Expression in TNBC Tumors

**Figure 5:**
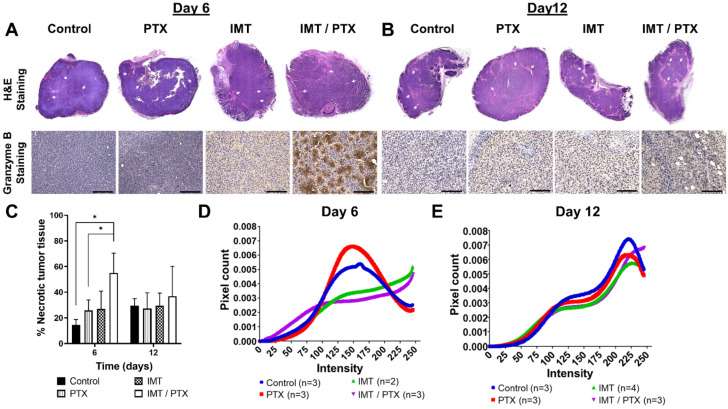
Histochemical imaging and quantitative analyses performed on E0771 tumors validates findings from analysis of granzyme B-specific positron emission tomography imaging data. Hematoxylin and eosin (H&E) staining and granzyme B expression in (**A**) day six tumors and (**B**) day 12 tumors are shown (20× magnification; scale: 125 µm). White arrows indicate necrotic regions. (**C**) Quantification performed on images (shows percentage of necrotic tissue from day six and 12 tumors. Mean ± SD (* indicates *p* < 0.05; unpaired *t*-test). Histogram analysis of pixel intensity in images of granzyme B staining on (**D**) day 6 and (**E**) day 12 tumors. Day six histograms: Control (blue) is different from paclitaxel (PTX), immunotherapy (IMT), and IMT/PTX distributions (*p* < 0.0001). IMT (green) is different from IMT/PTX distribution (purple) (*p* < 0.0001). Day 12 histograms: Control (blue) is different from PTX distribution (red; *p* < 0.006) and IMT (green) and IMT/PTX distributions (purple; *p* < 0.0001). Kolmogorov-Smirnov tests.

E0771 tumors showed consistent and higher sensitivity to IMT and combination IMT/PTX compared to 4T1 tumors (Figure 3) and less variation in quantitative analysis of GZP-PET images (Figure 4). Therefore, E0771 tumors were harvested on days six and 12 for early evaluation of biological changes in necrosis, granzyme B expression, and cytokine expression. Representative images of H&E staining showed more necrosis (light pink stain) in TNBC tumors treated with IMT alone and in combination with PTX compared to control and PTX-treated tumors (Figure 5A,B). Granzyme B staining showed more anti-granzyme B staining in combination PTX and IMT-treated tumors and IMT alone compared to the other treatment groups and control (Figure 5A). Quantitative analysis of H&E images for necrosis revealed that combination treatment with PTX and IMT resulted in 41% and 29% more necrotic tumor tissue, respectively, compared to control and IMT alone (*p* = 0.04, *p* = 0.01; Figure 5C) on day six; however, by day 12 (matching timing of reduction in tumor volume) the necrotic percentage was reduced (Figure 5C).

Histogram analysis of pixel intensity in images of tumors stained for granzyme B showed that PTX, IMT, and IMT/PTX had significantly different patterns of intensity compared to control tumor images on day six (*p* < 0.0001 for all comparisons; Figure 5D). Additionally, combination-treated tumor images had significantly different distributions of pixel intensity compared to images of IMT-only treated tumors (*p* < 0.0001; Figure 5D). On day 12, the distribution of control treated tumors was significantly different from that of single-agent PTX treated tumors (*p* < 0.006), IMT-only treated tumors (*p* < 0.0001), and combination IMT/PTX treated tumors (*p* < 0.0001; Figure 5E).

### 3.4. IMT plus PTX Increases IFN-γ, IL-2, and IL-12 Expression, and Th2 Response in E0771 Tumors

**Figure 6:**
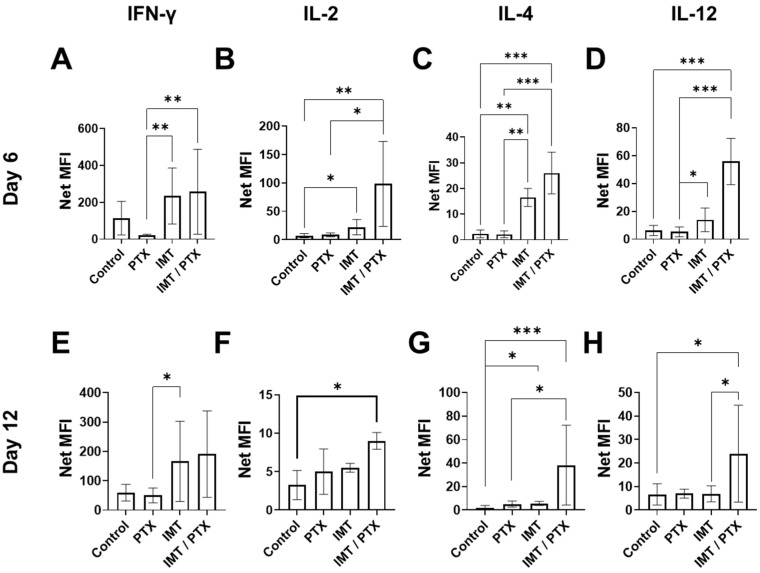
Combination immunotherapy/paclitaxel increases Th1/2 response to stimulate effector cell activation in E0771 tumors. E0771 tumors (*n* = 3/group) were extracted from mice on days 6 (**A**–**D**) and 12 (**E**–**H**) before significant changes in tumor volume. Mean ± SD (* indicates *p* < 0.05, ** indicates *p* < 0.001, *** indicates *p* < 0.0001; Dunn’s test). Abbreviations: Interleukin (IL), mean fluorescence intensity (MFI).

To understand the effects of combination chemotherapy and IMT, the expression levels of 11 Th1/Th2 soluble cytokines were measured from E0771 tumors extracted on days 6 and 12. Table 1 shows the expression of these cytokines in treated tumors as a percent of control tumor cytokine levels. Figure 6 shows differences between control and treatment groups in four key cytokines, IFN-γ, IL-2, IL-4, and IL-12, related to TNBC tumor response.

On day six, expression of IFN-γ was significantly increased by treatment with IMT alone (11 fold-change; *p* < 0.01) and in combination with PTX (12 fold-change; *p* < 0.005) compared to treatment with single-agent PTX (Figure 6A, Table 1). On day six, IMT-treated tumors had an IL-2 expression that was three times as much as control and combination IMT/PTX-treated tumors had an IL-2 expression that was 14 times as much as control (*p* = 0.02 and *p* < 0.01, respectively; Figure 6B). Compared to tumors treated with PTX alone, combination IMT/PTX tumors had a 10 fold increase in IL-2 expression (*p* = 0.03; Figure 6B). On day six, IL-4 expression was significantly increased by IMT alone (7 fold-change; *p* < 0.01) and in combination with PTX (11 fold-change; *p* < 0.0005) compared to control (Figure 6C). An 11 fold-change in IL-4 expression was observed when tumors were treated with combination IMT/PTX versus PTX alone (*p* < 0.0005; Figure 6C). A seven fold-change in IL-4 expression was observed when tumors were treated with IMT alone versus single-agent PTX (*p* < 0.001; Figure 6C). IL-12 expression was significantly increased by IMT alone (2 fold-change; *p* < 0.05) and with PTX (10 fold-change; *p* < 0.0005) compared to PTX alone on day 6 (Figure 6D). Compared to control, combination IMT/PTX-treated tumors had nine times more IL-12 (*p* < 0.0005; Figure 6D).

**Figure 7:**
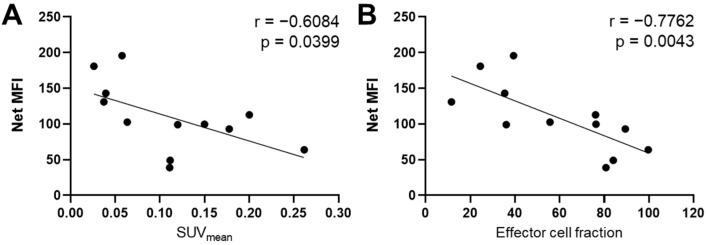
Tumor necrosis factor alpha (TNF-α) expression is negatively correlated with mean standard uptake value (SUV_mean_) and effector cell fraction. TNF-α expression from day 12 E0771 tumors were correlated with (**A**) SUV_mean_ and (**B**) effector cell fraction calculated from granzyme B-specific positron emission tomography imaging data on day six. Spearman’s rank-order correlation. Abbreviations: Mean fluorescence intensity (MFI).

By day 12, IMT increased IFN-γ expression by threefold compared to treatment with single-agent PTX (*p* = 0.03; Figure 6E). Additionally, by day 12, treatment with combination IMT/PTX resulted in significantly increased expression of IL-2 (3 fold-change; *p* = 0.03), IL-4 (24 fold-change; *p* < 0.0005), and IL-12 (4 fold-change; *p* = 0.02) compared to control (Figure 6F–H, Table 1). Compared to control, four times more IL-4 expression was observed in tumors treated with IMT alone by day 12 (*p* = 0.03; Figure 6G). Compared to tumors given PTX alone tumors, tumors given IMT/PTX had eight times more IL-4 expression by day 12 (*p* < 0.05; Figure 6G). Compared to tumors given IMT alone, combination-treated tumors had four times more IL-12 expression by day 12 (*p* = 0.03; Figure 6H). Lastly, a negative correlation between TNF-α expression on day 12 and granzyme B expression measured as SUV_mean_ (r = −0.61, *p* = 0.04; Figure 7A) and effector cell fraction (r = −0.78, *p* < 0.005; Figure 7B) on day six was found.

## 4. Discussion

In this study, we demonstrate that GZP-PET imaging provides early prediction of TNBC tumor response to the immune checkpoint blockade with chemotherapy. To the authors’ knowledge, this is the first study to evaluate the effect of immune checkpoint inhibition plus chemotherapy on effector cell activation with [^68^Ga]-NOTA-GZP in syngeneic, orthotopic mouse models of TNBC. The 4T1 model provides a secondary tumor model that supports GZP-PET imaging in response to combinatory therapy with IMT in breast cancer. Furthermore, as it is considered a poorly immunogenic model when compared with E0771 tumors, and this provides evidence of advanced imaging application to a wide range of response kinetics to IMT. We also investigate the direct effects of chemotherapy on granzyme B expression, which is clinically relevant as immunotherapy is often combined with other systemic treatments. We demonstrate that GZP-PET is able to provide quantitative information regarding effector cell activation in two models of TNBC tumors before physical changes in tumor volume can be measured and can be used to differentiate tumor response to treatment. Validation of these findings with IHC showed increased granzyme B in relationship with GZP-PET imaging data. Additionally, subsequent tumor analysis of soluble cytokine expression revealed that treatment with IMT plus PTX resulted in IFN-γ, IL-2, and IL-12 expression and Th2 response. These findings are significant because they contribute to our understanding of the biological impact that combination IMT and chemotherapy has on the TME.

As IMT becomes the standard-of care for many solid cancers, tracking immune cell infiltration and activity has become an important focus for monitoring the efficacy of IMT [18,19,34,43,44,45]. GZP-PET is one of several immunoPET-based imaging strategies for quantitatively predicting efficacious responses to cancer IMT. The present study provides evidence that GZP-PET imaging may be useful for rapid assessment of combination chemo- and immunotherapeutic efficacy in the clinical setting, particularly for TNBC patients. Notably, CD4+ and CD8a+ PET imaging with zirconium 89 has recently been studied to investigate the predictive value of T cell specific imaging [43,44]. Kristensen et al. found that the maximum [^89^Zr]-DFO-CD4 tumor-to-heart ratio could be used to stratify syngeneic mouse models, including 4T1, according to therapeutic response to Sym021, a humanized PD-1 antibody cross-reactive with mouse PD-1 [43]. Using a glioma murine model, Kasten et al. found that PET imaging with a [^89^Zr]-labeled anti-CD8 cys-diabody revealed CD8+ cell infiltration during oncolytic virus therapy. Additionally, others using non-imaging-based approaches have attempted to investigate the relationship between chemotherapy and the immune system [45,46]. Using flow cytometry to investigate chemotherapy-induced changes in sub-populations of immune cells, Onyema et al. found that immunosenescence appeared pronounced in patients with breast cancer and that the normal condition was not restored after six months of chemotherapy [45]. Furthermore, Samanta et al. show that when TNBC cells are treated with chemotherapy, the surviving cancer cells turn on genes that enable them to escape killing by the immune system [46,47,48]. Though the aforementioned studies support the evaluation of immune cells to understand treatment response, none evaluated the combination treatment with chemotherapy and IMT.

In congruence with similar studies [18,19,40,49], our investigation into the utility of granzyme B tracking with GZP-PET revealed that it is a useful, real-time molecular imaging approach for imaging the adaptive immune response and evaluating individual tumor response to treatment. It has been demonstrated that GZP-PET imaging is a promising method for measuring active antitumor immune response and for monitoring response to novel checkpoint inhibitors and combination regimens [18,19,50], and these results have initiated a clinical trial using GZP-PET imaging during treatment with IMT in melanoma and lung cancer (NCT04169321). Classification of tumors as responders based on eventual changes in tumor volume revealed granzyme B PET imaging to be highly sensitive and selective for treatment response [19]. Several studies have shown that TNBC immune checkpoint inhibitors are more effective in combination treatment than as a single agent, and recently, anti-PD1 has demonstrated promising results in early-stage TNBC [8,47]. However, none of these studies have evaluated early response to IMT in real-time on the molecular level using noninvasive imaging. Our study sought to analyze how immune checkpoint inhibition paired with chemotherapy affects tumor immune status. In addition to analysis of overall effector cell activation, distribution of effector cell activation, which is indicative of intratumoral heterogeneity, was observed through histogram analysis of radiotracer uptake in the TME. We found that by day six, treatment with IMT and PTX increased both the total amount and the distribution of effector cell activation compared to untreated control tumors, demonstrating a wider activation of effector cells across the tumor. These data on combination IMT/PTX-treated TNBC tumors are in congruence with previous findings that granzyme B secretion in the TME could serve as an early biomarker of response.

To further evaluate the underlying biological changes, molecular imaging findings were combined with histological staining, quantitative analysis of tumor immunohistochemistry, and analysis of soluble cytokine expression in homogenized tumors. As expected, we found the highest percentages of granzyme B expression in combination-treated tumors early in the course of IMT treatment, which corresponds with our imaging results. Though GZP binds intracellular caspases present in necrotic tissue, this binding occurs at much lower affinity than granzyme B; therefore, it was expected that IMT treatment would elicit apoptosis-mediated cell death compared to necrosis. Interestingly, we observed heightened IL-2, and IL-12 expression and Th2 response in E0771 tumors compared to control, indicating that immunogenic cell death (ICD) may have been induced as a result of combination IMT/PTX treatment [48]. These results support our hypothesis that combination IMT plus PTX would result in apoptosis from chemotherapy and enhanced effector cell response that would kill any remaining and/or PTX-resistant tumor cells. While ICD hallmarks such as high mobility group B1 and calreticulin were not directly observed in the present study, several important related mechanistic components were measured. In the ICD pathway, CD8^+^ T cells and TILs secrete IFN-γ to mediate antigen presentation and IL-2 is a well-known marker for T cells. Based on our findings, both IFN-γ and IL-2 expression were increased in E0771 tumors by treatment with combination IMT and PTX, indicating recruitment of T cells to the TME via the ICD pathway. IL-12 is a marker for Th1 response, and enhancement of Th1 effectors has been observed as an induction effect of ICD stimulation from anthracyclines [49]. Additionally, a correlation between IL-12 expression and granzyme B release was observed by LaSalle et al. [50]. Our study showed that IL-12 was increased in E0771 tumors by treatment with combination IMT and PTX, potentially stimulating local production of IFN-γ in the TME [51]. These results are important as other studies confirm increased apoptosis and chemotherapeutic treatment response in 4T1 cells through stimulation of T cell cytokine production, namely, IL-12 and IFN-γ [51,52,53].

The largest percent differences compared to control were observed in the expressions of IL-4, 5, and 13 in E0771 tumors. IL-4, 5, and 13 are markers for Th2 response and showed increased expression on days six and 12 due to combination treatment compared to control. Though Th2 response is not widely cited in ICD, it is of note that heightened Th2 response was observed in conjunction with IL-4, 5, and 13. This observation was opposite of our initial hypothesis because C57/Bl6 mice have been reported as having a genetically programmed bias toward Th1 immunity, mainly regulated by IFN-γ [54]. Th2 cytokines are associated with the presence of immunosuppressive cells in the TME, specifically myeloid-derived suppressor cells (MDSCs) and tumor-associated macrophages (TAMs) [55]. Additionally, Th2 cytokines, particularly IL-4, are associated with a humoral response and have been shown to promote tumor cell proliferation and resistance to apoptosis [55]. Therefore, Th1 stimulation by combination IMT/PTX treatment may be tampered by simultaneous Th2 stimulation. The negative correlation observed in E0771 tumors between TNF-α expression on day 12 and granzyme B on day six indicates that the cell-mediated (i.e., Th1) response is transient and not sustained by day 12 when physical changes in tumor volume due to IMT were noted. A recent study by Zhao et al. shows that exogenous TNF-α significantly reduced granzyme A, granzyme B, and perforin expression in specific subsets of CD8+ T cells [56]. The negative correlation we found supports data from this and other timely studies indicating that TNF inhibition may increase the efficacy of immune checkpoint inhibitors [57,58,59].

The present study is limited by its use of a simplified dose strategy and schedule for administration of IMT and chemotherapy. In our study, anti-PD1 and anti-CTLA4 were administered concurrently for the purpose of focusing our evaluation on response to combination PTX/IMT versus single-agent PTX and versus IMT (anti-PD1 and anti-CTLA4) without PTX. However, other studies have examined staggered dosing of these agents and many have demonstrated that timing of checkpoint inhibition is critical [60,61]. Other studies have also demonstrated that the benefits of immune checkpoint inhibition used in conjunction with chemotherapy occur at specific dose ranges [62,63]. Additionally, it is important to note that divergent tumor biology may contribute to conflicting Th1/Th2 cytokine profiles. Further experiments separating tumors into response categories based on treatment using advanced imaging strategies may provide more information on underlying biology. To better understand the relationship of imaging and cytokine release of effector cells, analysis of tumors with a wide range of IMT responses is important. Future work includes identifying timing, dosing, and dose sequencing strategies of anti-cancer therapies to increase effector cell activation in the TME as well as parsing response into partial and complete classifications. Assessment of specific immune cell phenotypes involved in the response to IMT and/or chemotherapy is also a primary focus of future investigation. Despite its current limitations, this study demonstrates the utility of GZP-PET as a novel approach to gaining insight on how the TME is altered by anti-cancer treatments. As more is understood about biological processes at the molecular and cellular levels within the breast cancer environment, molecular imaging with advanced PET techniques to assess early treatment response holds much promise for personalized therapy.

## 5. Conclusions

At this time, there are no clinically approved targeted therapies for TNBC; therefore, IMT in combination with chemotherapy (and other novel treatments) are being utilized in standard-of-care and explored heavily in clinical trials for various stages of disease. Additionally, there are no current predictive markers that robustly quantify who will benefit from IMT so that patient treatment regimens can be personalized to those who would show effective response. This project identifies an approach that could be used to personalize and optimize novel TNBC combination chemotherapy with IMT for individual tumors. This study is among the first to use quantitative imaging to assess the TNBC immune microenvironment and to examine the effects of combination chemotherapy and IMT for TNBC. Because breast cancer is typically treated with combination therapies, identifying effective treatment strategies and finding imaging biomarkers to assess combination therapy are key objectives. Results from this study show that noninvasive granzyme B-based PET imaging with [^68^Ga]-NOTA-GZP is a novel and valuable approach for measuring effector cell activation and predicting tumor response to combination chemotherapy and IMT in TNBC. Utilizing clinically-translatable advanced imaging strategies, such as GZP-PET, to understand the biologically distinct features of the TNBC TME can aid in personalizing anti-cancer therapies.

## Figures and Tables

**Table 1:** Paclitaxel (PTX), immunotherapy (IMT), or combination IMT/PTX resulted in differences in E0771 cytokine expression on days six and 12 before significant changes in tumor volume. Cytokine expression from mice given PTX, IMT, and IMT/PTX (*n* = 3/group) shown as % differences of baseline. Lowest values displayed in red; highest values displayed in green. Abbreviations: Granulocyte-macrophage colony-stimulating factor (GM-CSF), interferon gamma (IFN-γ), interleukin (IL), tumor necrosis factor alpha (TNF-α).

	DAY 6			DAY 12		
	PTX	IMT	IMT/PTX	PTX	IMT	IMT/PTX
GM-CSF	−2	−41	−12	−57	−78	−50
IFN-γ	−81	105	125	−17	182	237
IL-1β	−45	52	−56	13	78	77
IL-2	28	228	1366	54	69	172
IL-4	−13	588	983	400	418	3991
IL-5	22	463	999	11	686	1892
IL-6	−50	−81	−50	23	−56	−17
IL-12p70	−17	108	732	20	14	394
IL-13	61	524	1635	89	465	1770
IL-18	−32	25	−24	47	36	67
TNF-α	−24	−42	−13	−37	−49	−45

## Data Availability

Datasets and materials are available upon reasonable request to the corresponding author.
